# Influence of grasping postures on skin deformation of hand

**DOI:** 10.1038/s41598-023-48658-5

**Published:** 2023-12-05

**Authors:** Yanru Zhai, Shaoguo Wu, Qinyue Hu, Wenjing Zhou, Yue Shen, Xuefeng Yan, Yan Ma

**Affiliations:** School of Textile and Clothing, Nantong University, Nantong, 226019 China

**Keywords:** Anatomy, Health care, Health occupations, Engineering

## Abstract

To investigate the influence of different grasping postures on the hand’s skin deformation, a handheld 3D EVA SCANNER was used to obtain 3D models of 111 women in five postures, including a straight posture and grasping cylinders with various diameters (4/6/8/10 cm). Skin relaxation strain ratio (${\lambda }_{p}$) and surface area skin relaxation strain ratio (${\lambda }_{m}$) were used as measures of skin deformation between two landmarks and multiple landmarks, respectively. The effects of grasping posture on skin deformation in different directions were analyzed. The results revealed significant variations in skin deformation among different grasping postures, except for the width of middle finger metacarpal and the length of middle finger’s proximal phalanx. The ${\lambda }_{p}$ increased with decreasing grasping object diameter, ranging from 5 to 18% on the coronal axis, and from 4 to 20% on the vertical axis. The overall variation of ${\lambda }_{m}$ ranged from 5 to 37.5%, following the same trend as ${\lambda }_{p}$ except for the surface area of tiger’s mouth, which exhibited a maximum difference of 10.9% with significant differences. These findings have potential applications in improving the design of hand equipment and understanding hand movement characteristics.

## Introduction

Hand function plays a crucial role in the motor function of human limbs. The human hand possesses a complex physiological structure with over 20° of freedom^1^. However, due to the intricate physiological structure and powerful motor functionality, understanding and recognizing the grasping motion of the human hand is exceedingly challenging. Grasping movement causes skin deformation of the hand. The grasping posture is influenced by various factors, and the skin deformation varies with the grasping posture. Survey results indicated that while current hand appliances fulfill their intended functions, there is room for improvement in terms of user comfort^2^. This issue primarily arises because the production design of hand appliances predominantly relies on basic hand data, such as length and width, obtained when the hand is in a static posture, while the skin deformation of the hand is often overlooked in this process. Grasping occurs when an object maintains a consistent connection with the hand. Currently, research on grasping postures primarily involves robotic hands imitating human grasping^3–5^, and database-based grasping posture classification^6,7^, based on these studies, it can be found that the classification of grasping posture depends not only on the hand posture but also on the nature of contact between the hand and the object. Moreover, significant uncertainty remains regarding the impact of environmental factors on human grasping frequency^8,9^. This study focuses on investigating skin deformation by analyzing the thumb-aligned grasping posture, which is frequently observed in daily activities due to its high occurrence rate. Among the five fingers, the thumb holds significant importance as it opposes the other four fingers. The grasped object used for this study was the cylinder, which is commonly encountered in thumb-aligned grasping.

Human skin exhibits elasticity and undergoes deformation when subjected to various motion postures. Existing studies on skin deformation primarily rely on the utilization of 3D human models^10–13^, 2D planar segmentation measurement techniques^14–18^, and 3D scanning methods for landmark measurements^19,20^. The human hand comprises 8 wrist bones, 5 metacarpals, 14 finger bones, numerous muscles, and well-developed nerves^21^, which possesses an exceptionally high degree of freedom compared to other body parts, making it challenging to construct a 3D model for computing skin deformation. On the other hand, 2D planar segmentation measurement proves to be laborious and time-consuming when measuring multiple grasping postures. In contrast, data acquired through 3D scanning technology can be easily stored, with volume and perimeter measurement errors of 2.0% and 5.8%, respectively, falling within the acceptable range^22,23^.

Previous research utilizing three-dimensional scanning technology primarily concentrated on areas such as the shoulders^24^, legs^25,26^, and other regions characterized by extensive deformation and limited degrees of freedom. However, the intricate structure of the hand adds complexity to the study of skin deformation, resulting in comparatively fewer investigations in this area. In a relevant study, Nasir et al.^27^ employed 3D scanning technology to assess skin deformation in the human hand across three postures from the perspective of designing therapeutic gloves. While their analysis identified deformation differences within the hand across these postures, they did not delve into the specific deformation patterns arising from different grasping postures. Nevertheless, their work holds valuable reference significance for this study.

In this study, a novel approach that employed a handheld 3D scanner to measure landmark distances across various grasping postures was introduced. Specifically, the investigation focused on the thumb alignment grasping posture, which commonly occurs during everyday activities due to its high frequency. The primary objective was to analyze the resulting skin deformation in the hand. By understanding the distribution of skin deformation at different grasping postures, designers can optimize the flexibility of hand appliances to mimic natural hand movements more accurately, which can enhance the comfort of wearing and improve overall functionality. The data and findings derived from this study can serve as a reference for deepening our comprehension of hand skin deformation characteristics and have significant implications for designing hand equipment aimed at enhancing safety protection.

## Methods

### Equipment

A non-contact handheld 3D EVA SCANNER (USA, Artec Studio) was used to capture 3D scanning models of the human hand. The scanner had an accuracy within 0.1 mm and a resolution within 0.2 mm. In this study, the scanner was solely used to generate a point cloud dataset of 3D objects (hands). The scanning speed was 16 fps, and the scanned image was saved in STL format. Following the scanning process, the data was processed using Artec Studio software. Subsequently, the original point cloud data was imported into Geomagic Studio (USA, Triangle Development Zone, North Carolina).

### Subjects

A total of 111 physically healthy female volunteers, aged between 18 and 26 years, were recruited for this study. All volunteers were Chinese. Only right hands were included in the collected 3D scan models, and the ranges of hand length and palm width were: 155–205 mm and 65–95 mm, respectively. None of the subjects exhibited severe injuries or obvious trauma to the right hand.

### Experimental procedure

The experiment was conducted in accordance with the general requirements for 3D scanning anthropometric methodologies (ISO 20685:2005, mod), and the experimental protocol was approved by the licensing committee of Textile and Clothing College of Nantong University.

**Figure 1:**
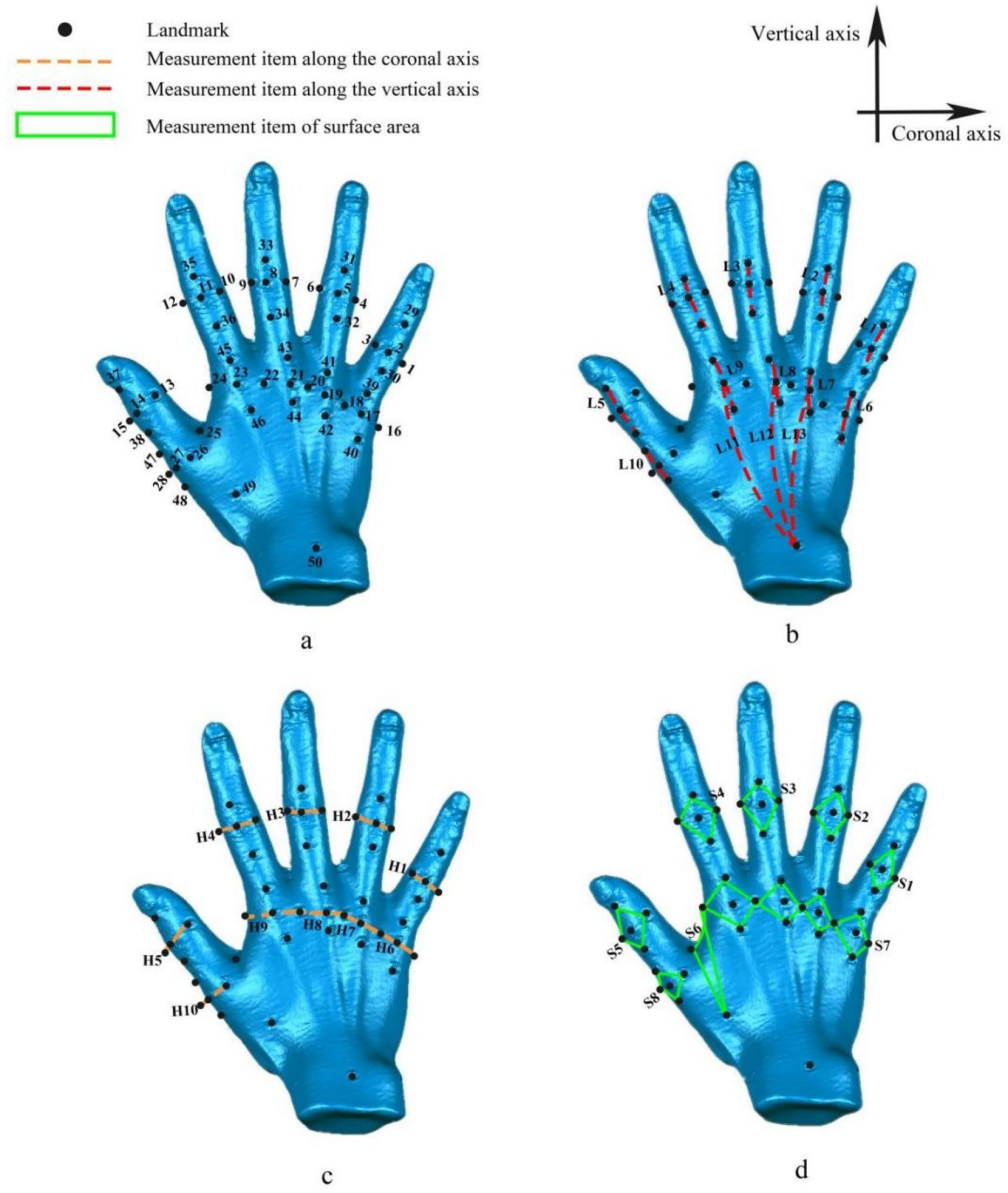
The positions of landmarks and the corresponding measurement items. (**a**) Diagram of the landmarks positions on the hand, (**b**) measurement items on the vertical axis, (**c**) measurement items on the coronal axis, (**d**) measurement items of the surface area.

Prior to the start of the experiment, each subject provided informed consent and signed the necessary documents alongside the scanner. Before scanning, it was ensured that the subject's right hand was free of any additional jewelry. Alcohol wipes were used to remove sweat and oil from the hands to ensure secure attachment of landmark stickers. Once the subjects' hands were dry, 4 mm diameter and 0.1 mm thick landmarks were placed on predetermined locations of the right hand of each subject (refer to Fig. 1a).

**Figure 2:**
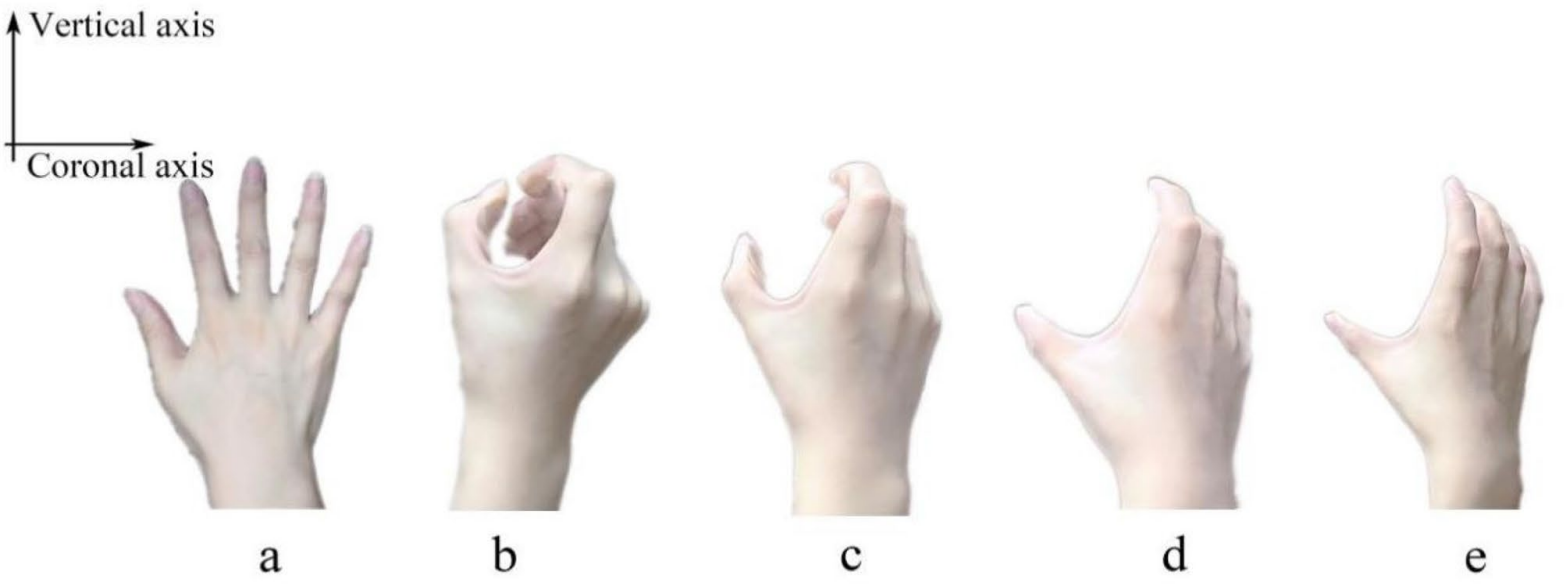
Schematic diagram of scanning posture. (**a**) Posture 0: straightening posture, (**b**) posture 1: grasp a cylinder with d = 4 cm, (**c**) posture 2: grasp a cylinder with d = 6 cm, (**d**) posture 3: grasp a cylinder with d = 8 cm, (**e**) posture 4: grasp a cylinder with d = 10 cm.

**Figure 3:**
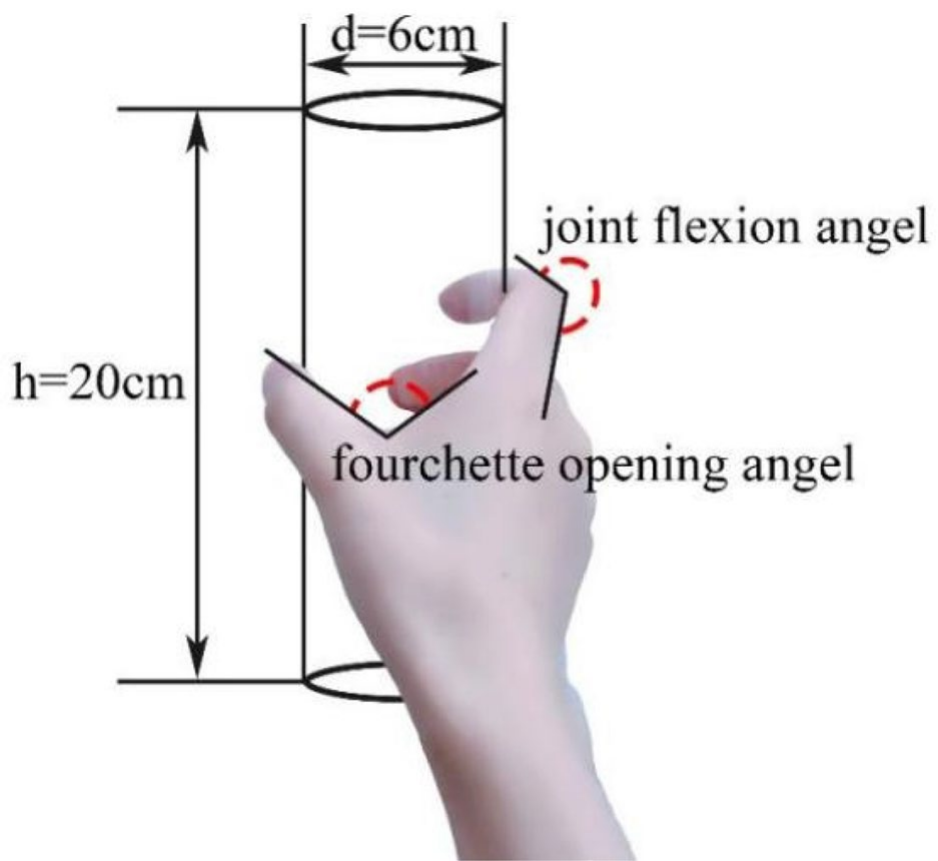
Diagram of joint flexion when grasping a cylinder.

Based on the recognition that human hand skin deformation is intricate, restricting the scanning postures would aid in comprehending the underlying principles of skin deformation. As mentioned, cylinder was selected as the grasping object due to the high frequency of use in industrial production processes^3^. The entire experiment was divided into five phases of posture scanning, including a straight posture and grasping transparent cylinders with diameters of 10 cm, 8 cm, 6 cm, and 4 cm (see Fig. 2), and all transparent cylinders were of the same height. No additional force was applied during the grasping. Only the upper surface of the subject's hand was scanned and analyzed during the process. The five postures were completed consecutively. All subjects underwent a standardized set of experimental procedures and postural training. In Fig. 2a, in order to maintain a consistent level of hand extension for each subject and to prevent errors caused by excessive forward leaning or bending of the hand, the subject's hand had to be straightened and affixed to the transparent glass plate before creating the 3D scan model of the hand. All grasping postures (refer to Fig. 2b–e) were executed using the thumb alignment grasping posture to minimize potential errors resulting from variations in grasping postures during the experiment. To accurately describe the trend of skin deformation in the hand, the angle formed when the proximal phalangeal joint is flexed medially towards the palm is defined as the joint flexion angle, the fourchette opening angle represents the angle formed by the thumb and index finger (see Fig. 3).

### Measurement items

The areas of the hand where skin deformation occurs due to the grasping posture need to be thoroughly studied. The measured items were categorized into surface area measurement and distance measurement on both vertical and coronal axes, taking into account the characteristics of the skin deformation when the grasping posture varied. Table 1 lists the names of the measurement items and landmarks. Figure 1b–d highlights the corresponding areas of the measurement items.

**Table 1:** Classification of measurement items and landmark names.

Hand region	Direction	Landmark name				
Proximal phalanx area	Vertical	L1	L2	L3	L4	L5
	Coronal	H1	H2	H3	H4	H5
	Surface area	S1	S2	S3	S4	S5
Metacarpal area	Vertical	L6	L7	L8	L9	L10
	Coronal	H6	H7	H8	H9	H10
	Surface area	S7	S8	–	–	–
Dorsum area	vertical	L11	L12	L13	–	–
Tiger’s mouth	Surface area	S6	–	–	–	–

## Data processing

### Model restoration

After removing point cloud data unrelated to the subject’s hand model, only the right-hand 3D point cloud data was retained in Artec Studio. The retained point cloud data underwent processing steps such as overall alignment, outlier noise removal, sharp fusion, and small object filtering. These steps aim to obtain an incompletely closed hand 3D scan model. The obtained model was then imported into Geomagic Studio for full filling, resulting in a complete 3D scanned model of the hand that can be measured. Multiple scanned models were collected for each hand posture, and the scanned model with the best scan fidelity was chosen as the data acquisition model. The “Draw Curve” tool in Geomagic Studio was utilized to measure the skin deformation of the hand between two landmarks in different directions, each measurement was repeated three times, and mean values were calculated.

### Skin relaxation strain ratio calculation

Introducing the concept of skin relaxation strain ratio proposed by Yanzhen Wang et al.^26^ The skin relaxation strain ratio between the two landmarks is:

1 $${\lambda }_{p}=\frac{b-a}{a}\times 100\%$$

The surface area skin relaxation strain ratio between landmarks is:

2 $${\lambda }_{m}=\frac{d-c}{c}\times 100\%$$

where ${\lambda }_{p}$ (%) represents the skin relaxation strain ratio between two landmarks; *b* (mm) represents the distance between two landmarks in a particular grasping posture; *a* (mm) represents the distance between two landmarks in the straight posture. ${\lambda }_{m}$ (%) represents the relaxation strain ratio of the skin surface area of the hand; *d* (mm^2^) represents the skin surface area of the hand in a particular grasping posture; *c* (mm^2^) represents the skin surface area of the hand in the straight posture.

### Normality test

Statistical software for social sciences (SPSS version 25) was used to analyze the skin relaxation strain ratio. The K–S test was used to determine the normality of the data distribution. It is appropriate for verifying the normality of a large data sample defined as a large sample with more than 50 rows of data. The 111 valid rows of data in this study made the normality test findings from the K–S test credible. The K–S test compares the frequency distribution $f\left(x\right)$ with the theoretical distribution $g\left(x\right)$ or the distribution of two observations. The null hypothesis H_0_ states that: the two data sets are normally distributed or consistent with the theoretical distribution. $D=max|f\left(x\right)-g\left(x\right)|$, when the actual observation $D>D\left(n,a\right)$, H_0_ is rejected, otherwise, the H_0_ hypothesis is not rejected. In this study, the K–S test was employed to determine if there are statistically significant differences in hand surface deformation exist among the grasping postures at each measurement item to further determine whether our data conformed to a normal distribution, which is a prerequisite for making valid inferences to larger populations based on our sample. From Table 2, it can be observed that *p* ≤ 0.05 for all sites except H8 (*p* = 0.200) and L3 (*p* = 0.063), which indicates that the H_0_ was rejected, suggesting that there were statistical differences between the different postures of the individual parts of the hand.

**Table 2:** Normality test for each part of the hand.

Measurement item	K–S normality test		S–W normality test	
	Statistics	Significance	Statistics	Significance
H1	0.118	0.000	0.881	0.000
H2	0.103	0.000	0.905	0.000
H3	0.096	0.000	0.875	0.000
H4	0.122	0.000	0.823	0.000
H5	0.106	0.000	0.904	0.000
H6	0.147	0.000	0.851	0.000
H7	0.090	0.000	0.927	0.000
H8	0.035	0.200	0.985	0.000
H9	0.075	0.000	0.931	0.000
H10	0.101	0.000	0.932	0.000
L1	0.145	0.000	0.703	0.000
L2	0.066	0.000	0.972	0.000
L3	0.042	0.063	0.985	0.000
L4	0.097	0.000	0.948	0.000
L5	0.098	0.000	0.899	0.000
L6	0.058	0.001	0.980	0.000
L7	0.060	0.001	0.962	0.000
L8	0.094	0.000	0.945	0.000
L9	0.120	0.000	0.876	0.000
L10	0.090	0.000	0.934	0.000
L11	0.199	0.000	0.557	0.000
L12	0.283	0.000	0.289	0.000
L13	0.272	0.000	0.300	0.000
S1	0.114	0.000	0.879	0.000
S2	0.085	0.000	0.946	0.000
S3	0.081	0.000	0.959	0.000
S4	0.095	0.000	0.917	0.000
S5	0.106	0.000	0.916	0.000
S6	0.091	0.000	0.924	0.000
S7	0.054	0.003	0.979	0.000
S8	0.308	0.000	0.278	0.000

## Results and discussion

### Skin deformation analysis on the coronal axis

**Figure 4:**
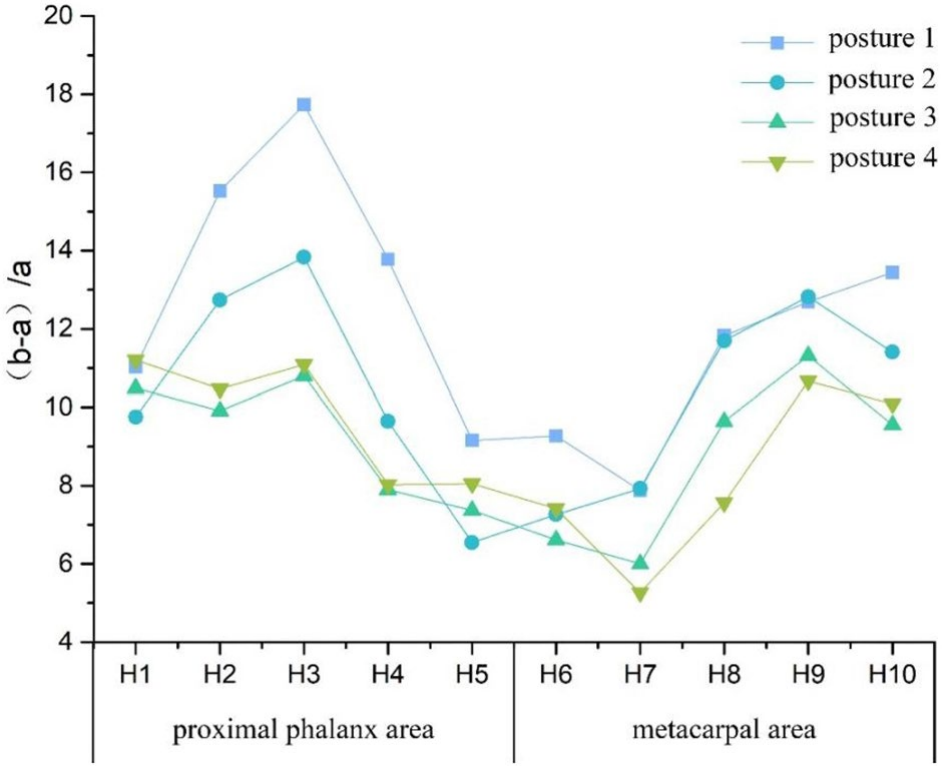
Folding graph of skin relaxation strain ratio on the coronal axis at different grasping postures (%).

To investigate the skin deformation on coronal axis during different grasping postures, the ${\lambda }_{p}$ on the coronal axis in different parts of the hand when grasping transparent cylinders with diameters of 4/6/8/10 cm was compared and analyzed. The results were shown in Fig. 4. The deformation area with the amount of change within 5% was regarded as the area with insignificant change^16^. The ${\lambda }_{p}$ changes on the coronal axis ranged from 5 to 18%. When the grasping objects had the same shape, the trend of ${\lambda }_{p}$ variation was generally similar, with the skin deformation gradually increasing as the diameter of the grasping objects decreased. Compared to the metacarpal area, the proximal phalangeal area exhibited slightly more skin deformation. In the proximal phalangeal area, the variation of ${\lambda }_{p}$ ranged from 6 to 18%, with the largest skin deformation observed in H3 during posture 1, with ${\lambda }_{p}$ of 17.2%, followed by postures 2, 3, and 4, with ${\lambda }_{p}$ of 13.8%, 10.8%, and 11.1%, respectively. The smallest skin deformation was found in H5, with a maximum difference of ${\lambda }_{p}$ less than 5% observed in the four grasping postures, indicating that the difference in skin deformation in this area was not significant. The ${\lambda }_{p}$ at H1, H2 and H3 was generally higher than those at H4 and H5. In the metacarpal region, the variation of ${\lambda }_{p}$ ranged from 5 to 14%, with the largest skin deformation observed in H10 during posture 1, with ${\lambda }_{p}$ of 13.4%, followed by postures 2, 3, and 4, with ${\lambda }_{p}$ of 11.4%, 10.1%, and 9.5%, respectively. In contrast to the skin deformation trend in the proximal phalangeal region, the ${\lambda }_{p}$ of deformation was generally higher at H8, H9, and H10 than at H6 and H7.

The reasons for skin deformation in the proximal phalangeal area and the metacarpal area were not the same when grasping cylindrical objects. In the proximal phalangeal area, the flexion of the proximal phalangeal joints led to skin deformation on both sides of the joints along coronal axis. The flexion of the proximal phalangeal joints of the little finger, ring finger and middle finger resulted in larger skin deformation on the coronal axis, while the proximal phalangeal joints of the index finger and thumb only played a smaller role in skin deformation due to their smaller flexion angles. In the metacarpal area, skin deformation on the coronal axis depended largely on the fourchette opening angle. A greater fourchette opening angle resulted in more stretching of the skin at the base of the finger to both sides, leading to greater skin deformation at the metacarpal joint. To maintain a stable grip, the fourchette opening angle increased of the thumb, index finger, and middle fingers, resulting in increased skin deformation at the metacarpal joint. Conversely, the fourchette opening angle was smaller for the little finger and ring finger, resulting in less skin deformation on the coronal axis.

### Skin deformation analysis on the vertical axis

**Figure 5:**
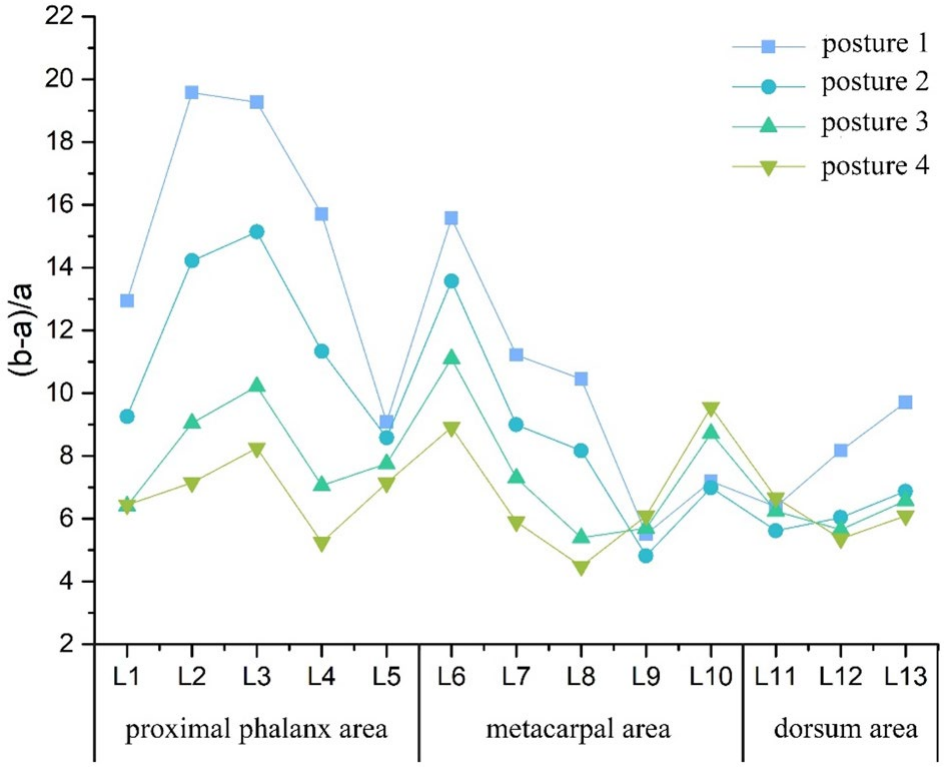
Folding graph of skin relaxation strain ratio on the vertical axis at different grasping postures (%).

In order to compare and analyze the skin deformation on the vertical axis caused by different grasping postures of the hand, the results of the comparison were shown in Fig. 5. Obviously, the overall ${\lambda }_{p}$ variation in skin deformation on the vertical axis ranged from 4 to 20%, which exhibited a similar trend to the ${\lambda }_{p}$ variation on the coronal axis. This means that the same shape of the grasped object resulted in a consistent pattern of ${\lambda }_{p}$ variation in skin deformation, with the ${\lambda }_{p}$ increasing as the diameter of the objects decreased within the same area. Specifically, the skin deformation in the proximal phalangeal area was slightly higher than that the metacarpal area. Within the proximal phalangeal area, the variation of ${\lambda }_{p}$ ranged from 5 to 20%. Notably, the skin deformation was more pronounced in L2 and L3 across all four grasping postures, with ${\lambda }_{p}$ of 19.6% and 19.3% respectively in posture 1, followed by postures 2, 3, and 4. On the other hand, the skin deformation at L5 relatively smaller, ${\lambda }_{p}$ ranging from 7 to 9%, and no significant difference was observed among the four grasping postures. In the metacarpal area, ${\lambda }_{p}$ varied from 4 to 16%, with L6 producing the largest skin deformation across all four grasping postures: 15.7%, 13.6%, 11.1% and 8.9%, respectively. The skin deformation at L9 and L10 was comparatively smaller, with ${\lambda }_{p}$ ranging from 4 to 10%. In the dorsal area, the skin deformation was less pronounced, with ${\lambda }_{p}$ ranging from 6 to 10%, and no significant difference was observed among the grasping postures.

During grasping, the skin deformation on the vertical axis resulting from flexion of the proximal phalangeal joint and metacarpal joint was slightly higher than the skin deformation on the coronal axis. Within the proximal phalangeal area, the ${\lambda }_{p}$ on the vertical axis exhibited a similar distribution to that on the coronal axis, which can be attributed to the same factors causing skin deformation in both directions at the proximal phalangeal joints. The proximal phalanx area of the little, ring, and middle fingers played a dominant role in grasping the cylinder, where an increase in joint flexion angle led to greater deformation on the vertical axis. The index finger and thumb had a fixed role with smaller joint flexion angles, resulting in less deformation on the vertical axis. In the metacarpal area, the distribution of ${\lambda }_{p}$ on the vertical axis differed slightly from that on the coronal axis. The extent of skin deformation on the vertical axis in this area largely depended on the joint flexion angle, meaning that a greater joint flexion angle of the metacarpal joint resulted in more pronounced deformation. The metacarpal area of the little, the ring and the middle fingers played a role in stabilizing the grasped object when gripping the cylinder, leading to an increased joint flexion angle of the metacarpal and a decrease in the fourchette opening angle. As a result, the skin deformation on the vertical axis was larger while the deformation on the coronal axis was smaller. In the dorsal area, due to the interconnectedness of the hand’s skin, flexion of the metacarpal joint caused stretching of the skin at that joint, resulting in similar skin deformation throughout the metacarpal palm. However, compared to the skin deformation at the metacarpal joint, the skin deformation in the dorsal area was smaller.

### Surface area skin deformation analysis

**Figure 6:**
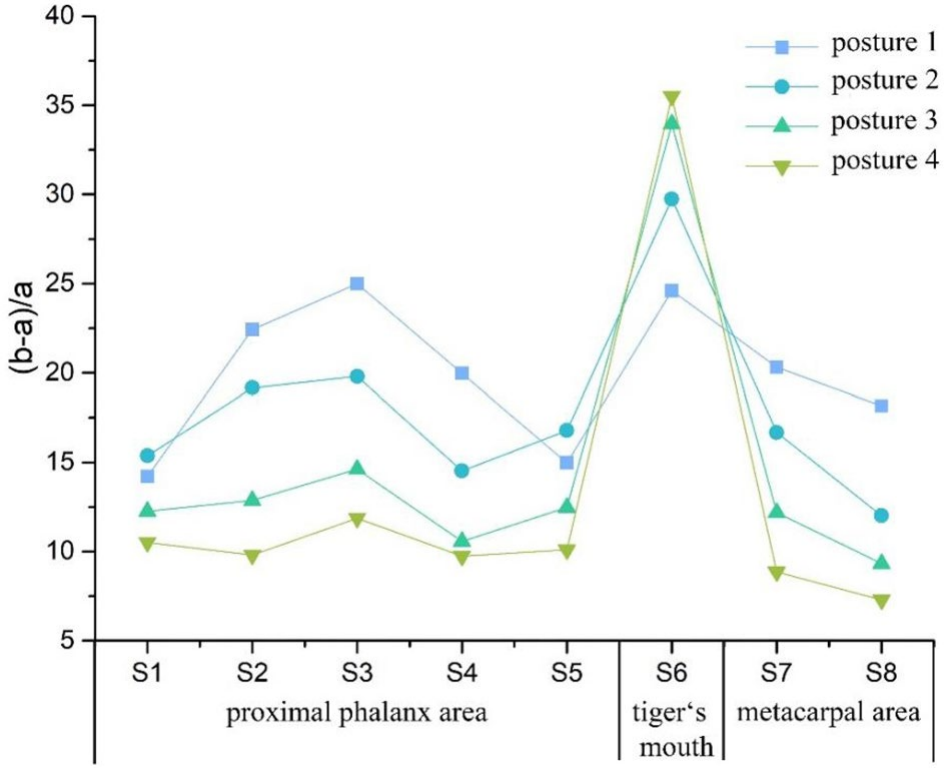
Folding graph of surface area skin relaxation strain ratio at different grasping postures (%).

To obtain a comprehensive understanding of the skin deformation across different areas of the surface, Fig. 6 compared and analyzed the distribution of ${\lambda }_{m}$ at various grasping postures. The results revealed that the highest degree of skin deformation occurred at S6, with ${\lambda }_{m}$ of 35.5%. The overall range of ${\lambda }_{m}$ varied between 5% and 37.5%, with all other parts (except S6) exhibiting a similar pattern as ${\lambda }_{p}$. Specifically, within each part, posture 1 resulted in the greatest skin deformation, followed by postures 2, 3, and 4 in sequential order. In the proximal phalangeal area, ${\lambda }_{m}$ and ${\lambda }_{p}$ displayed identical distribution patterns, the overall variation of ${\lambda }_{m}$ ranged from 9 to 25%. Notably, S3 exhibited the largest skin deformation in posture 1, with ${\lambda }_{m}$ of 25%, followed by 19.8%, 14.6% and 11.9% for subsequent postures. S2 and S4 demonstrated slightly lower levels of skin deformation compared to S3. Regarding the metacarpal area, ${\lambda }_{m}$ ranged from 7 to 21%, slightly smaller than that observed in the proximal phalangeal area. Surprisingly, S6 exhibited an inverse distribution pattern of ${\lambda }_{p}$ compared to other areas, with the lowest skin deformation occurring in posture 1 and the highest in posture 4. The maximum difference of ${\lambda }_{m}$ in this area across the four postures was 10.9%, indicating significant variations in skin deformation due to different grasping postures.

The tiger's mouth, referring to the area between the index finger and the thumb, experienced the most significant skin deformation during the grasping process. This was primarily due to the shape limitation of the grasping object, where the index finger and thumb opposed each other. As the diameter of the grasping cylinder increased, the fourchette opening angle between the index finger and thumb also increased. Consequently, the skin at the tiger's mouth was stretched, resulting in the opposite distribution of skin deformation compared to other areas. In the proximal phalangeal area, greater skin deformation was observed on the middle finger and the ring finger during grasping action. This indicated that the joint flexion angle of the proximal phalangeal joint increased more in these fingers compared to others. In the metacarpal area, the skin deformation exceeded 5%, which was a combination of both vertical and coronal axis deformations.

## Conclusion

1. The skin deformation at each measurement area of the hand was significantly different under different grasping postures, except for H8 and L3.
2. The ${\lambda }_{p}$ variation in the proximal phalangeal area and the metacarpal area was generally consistent with the same shape of the grasped object, and the ${\lambda }_{p}$ in the same area increased gradually with decreasing grasping object diameter. The skin deformation caused in the proximal phalangeal area was slightly higher than that in the metacarpal area.
3. The ${\lambda }_{p}$ on the coronal axis ranged from 5 to 18%, and from 4 to 20% on the vertical axis. The ${\lambda }_{p}$ at the dorsum area ranged from 6 to 10%, and there was no significant difference among the four grasping postures at the dorsum area.
4. The overall variation of ${\lambda }_{m}$ ranged from 5% to 37.5%. ${\lambda }_{m}$ in the metacarpal area was slightly smaller than that in the proximal phalangeal area. S6 showed the opposite distribution of ${\lambda }_{p}$, the ${\lambda }_{m}$ of S6 decreased gradually with the decrease of the diameter of the grasped object, and there were significant differences in ${\lambda }_{m}$ in this area among the four grasping postures.

## Data Availability

As the data will be used in the development and design of protective gloves, the dataset for the period of this study is not publicly available, but is aquired from the corresponding author upon reasonable request.
